# Unraveling the Specific Ischemic Core and Penumbra Transcriptome in the Permanent Middle Cerebral Artery Occlusion Mouse Model Brain Treated with the Neuropeptide PACAP38

**DOI:** 10.3390/microarrays4010002

**Published:** 2015-01-21

**Authors:** Motohide Hori, Tomoya Nakamachi, Junko Shibato, Randeep Rakwal, Seiji Shioda, Satoshi Numazawa

**Affiliations:** 1 Division of Toxicology, Department of Pharmacology, Toxicology and Therapeutics, School of Pharmacy, Showa University, 1-5-8 Hatanodai, Shinagawa, Tokyo 142-8555, Japan; E-Mails: author3cityform@yahoo.co.jp (M.H.); numazawa@pharm.showa-u.ac.jp (S.N.); 2 Department of Anatomy, School of Medicine, Showa University, 1-5-8 Hatanodai, Shinagawa, Tokyo 142-8555, Japan; E-Mails: nakamachi@med.showa-u.ac.jp (T.N.); rjunko@nifty.com (J.S.); 3 Laboratory of Regulatory Biology, Graduate School of Science and Engineering, University of Toyama, Toyama, Toyama 930-8555, Japan; 4 Laboratory of Exercise Biochemistry and Neuroendocrinology, Institute of Health and Sports Sciences, University of Tsukuba, Tsukuba, Ibaraki 305-8574, Japan; 5 Organization for Educational Initiatives, University of Tsukuba, 1-1-1 Tennoudai, Tsukuba, Ibaraki 305-8577, Japan

**Keywords:** ischemic core, penumbra, PACAP38, differential gene expression, *Ahsp*, *Ttr*

## Abstract

Our group has been systematically investigating the effects of the neuropeptide pituitary adenylate-cyclase activating polypeptide (PACAP) on the ischemic brain. To do so, we have established and utilized the permanent middle cerebral artery occlusion (PMCAO) mouse model, in which PACAP38 (1 pmol) injection is given intracerebroventrically and compared to a control saline (0.9% sodium chloride, NaCl) injection, to unravel genome-wide gene expression changes using a high-throughput DNA microarray analysis approach. In our previous studies, we have accumulated a large volume of data (gene inventory) from the whole brain (ipsilateral and contralateral hemispheres) after both PMCAO and post-PACAP38 injection. In our latest research, we have targeted specifically infarct or ischemic core (hereafter abbreviated IC) and penumbra (hereafter abbreviated P) post-PACAP38 injections in order to re-examine the transcriptome at 6 and 24 h post injection. The current study aims to delineate the specificity of expression and localization of differentially expressed molecular factors influenced by PACAP38 in the IC and P regions. Utilizing the mouse 4 × 44 K whole genome DNA chip we show numerous changes (≧/≦ 1.5/0.75-fold) at both 6 h (654 and 456, and 522 and 449 up- and down-regulated genes for IC and P, respectively) and 24 h (2568 and 2684, and 1947 and 1592 up- and down-regulated genes for IC and P, respectively) after PACAP38 treatment. Among the gene inventories obtained here, two genes, brain-derived neurotrophic factor (*Bdnf*) and transthyretin (*Ttr*) were found to be induced by PACAP38 treatment, which we had not been able to identify previously using the whole hemisphere transcriptome analysis. Using bioinformatics analysis by pathway- or specific-disease-state focused gene classifications and Ingenuity Pathway Analysis (IPA) the differentially expressed genes are functionally classified and discussed. Among these, we specifically discuss some novel and previously identified genes, such as alpha hemoglobin stabilizing protein (*Ahsp*), cathelicidin antimicrobial peptide (*Camp*), chemokines, interferon beta 1 (*Ifnb1*), and interleukin 6 (*Il6*) in context of PACAP38-mediated neuroprotection in the ischemic brain. Taken together, the DNA microarray analysis provides not only a great resource for further study, but also reinforces the importance of region-specific analyses in genome-wide identification of target molecular factors that might play a role in the neuroprotective function of PACAP38.

## 1. Introduction

Working on brain ischemia [1] and the neuroprotective effects of pituitary adenylate-cyclase activating polypeptide (PACAP) [2,3,4,5,6,7,8,9,10,11], we have previously reported the establishment of a permanent middle cerebral artery occlusion (hereafter abbreviated PMCAO) mouse model, and our results on the genome-wide transcriptomic profile in the whole brain/hemisphere [12,13,14]. These results revealed numerous gene expressions that were being modulated in the ischemic brain with or without PACAP38 treatment, providing a first inventory of the mouse ischemic brain transcriptome [12,13,14]. To note, in our group, we have been using the intraluminal filament technique-based PMCAO model as compared to the transient middle cerebral artery occlusion (MCAO), which results in reperfusion injury that we wished to avoid in our research model. Nevertheless, different groups have used different stroke models including the MCAO, which has resulted in providing a broader insight into the PACAP effects on the ischemic brain.

Previous research on endogenous and exogenous PACAP neuroprotective effects using animal models has provided enormous data to suggest the potential therapeutic use of PACAP38 and/or its analogs. Looking specifically at the PACAP action in stroke models—the focus of our group’s research—the data have revealed not only the genes involved in ischemia but also novel molecular factors in the ischemic brain and potential mechanisms therein [15,16,17,18]. For example, Tamas and co-workers, using the rat PMCAO model, showed that a single bolus injection of PACAP but not vasoactive intestinal polypeptide (VIP) reduced the infarct size slowing down progression of the evolution of infarct, subsequently reducing final infarct size, under the same experimental conditions [15]. On the other hand using a MCAO model, Chen and co-workers elegantly demonstrated that PACAP38 administered intravenously (i.v.) or intracerebroventrically (i.c.v.) 1 h after the occlusion significantly reduced infarct volume including an improvement in functional motor deficits after 24 h in wild-type mice [16]. Those authors also performed a cDNA microarray-based transcriptome profiling revealing a critical role for PACAP in later response injury over the early response. Recently, in 2011, Dejda and co-workers further showed that PACAP and a novel stable analog were helpful in protecting rat brain against ischemia using a MCAO model by not only suppressing the apoptotic response but by an ability to modulate the inflammatory responses [17]. Most recently, using the PACAP-deficient mice for understanding the endogenous protective effects of the neuropeptide, Reglodi and co-workers summarized data indicating the vulnerability of these mice to diverse *in vitro* and *in vivo* insults [18]. From their study, it can be indicated that endogenous PACAP is not only an in-built critical factor in protecting the brain and nervous system from injuries including ischemia but also in peripheral organs such as the large intestine, pancreas, and the heart. As also recently reviewed by Tamas and co-workers in 2012, the role of PACAP as a neurotrophic factor has been validated and data suggesting an important function of PACAP in neuronal regeneration promises its use as a therapeutic agent in injuries to the nervous system [19].

PACAP38 is undoubtedly a neuroprotective agent in ischemia as evidenced by our and other groups’ research using animal models (for examples, [12,13,14,15,16,17,18,19], and references therein). Despite these studies that have contributed enormously to both the possible mechanism/s behind PACAP38 action and underlying molecular factors and/or pathways, the target has been the whole brain or the hemispheres. Most recently, working with the hypothesis that targeting the infarct or ischemic core (hereafter abbreviated IC) and penumbra (hereafter abbreviated P) specifically would reveal more specific gene candidates in these two regions of the ischemic brain, we recently performed a new experiment at 6 and 24 h post-ischemia after dissecting out the IC and P (in the ischemic hemisphere) from PMCAO mouse model brains [20]. To check our hypothesis on the excised samples, we performed traditional reverse transcription-polymerase chain reaction (RT-PCR) analysis on selected candidate genes and Western blot analysis for a protein candidate—collapsin response mediator protein 2 (CRMP2)—based on our previous findings [12,13,14]. Those results revealed a distinct expression profile for each of the selected genes and the CRMP2 protein in both IC and P regions [20]. These expression profiles for genes (mRNA level) and the CRMP2 protein (protein abundance) were found to be different from that observed during previous studies using the whole hemispheres [12,13,14], indicating the importance of targeted brain region analysis for a better insight into the effect of PACAP38 on the genome and the proteome.

With this supporting information and confidence in our hypothesis, we proceeded to utilize the highly expensive, time-consuming but informative omics analysis, namely the DNA microarray approach [21], in this present study to specifically target these two regions (IC and P) within the ischemic hemisphere. Thus the aim of this present study is to identify and/or further narrow down the genes that may be specifically related to the neuroprotective action of PACAP38 region-wise and delineate the gene expression at the initial (6 h post-PACAP38 injection) and later (24 h post-PACAP38 injection) phases of response. Our results reveal a more specific (compared to our whole hemisphere studies) PACAP38 influenced IC and P transcriptome at both initial (6 h) and later (24 h) time points post-treatment, simultaneously functionally categorizing the PACAP38-regulated genes according to their biological roles based on additional bioinformatics analyses.

## 2. Results and Discussion

### 2.1. Experimental Design for Targeted Brain Region Dissection of Ischemic Core (IC) and Penumbra (P), Total RNA Extraction and Quality Check, cDNA Synthesis and RT-PCR, and DNA Microarray Analysis

**Figure 1:**
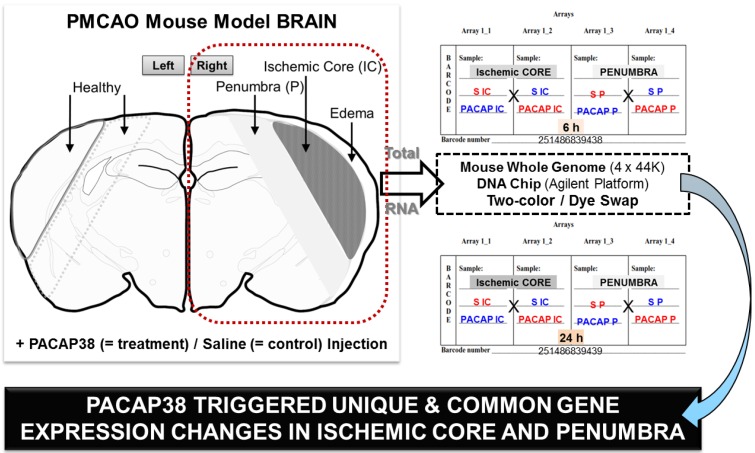
Experimental strategy for genome-wide analysis of the ischemic core (IC) and penumbra (P) transcriptome following pituitary adenylate-cyclase activating polypeptide (PACAP38) treatment. Diagrammatic representation of the permanent middle cerebral artery occlusion (PMCAO) model (left-hand side). Total RNA was extracted from the IC and P in the ipsilateral (right) hemisphere along with the corresponding regions in the healthy contralateral hemisphere. PACAP38 was the treatment and saline served as the control. DNA microarray analysis was performed using two oligo DNA chips (Agilent Technologies) as indicated using the two-color, dye-swap approach (right-hand side).

**Figure 2:**
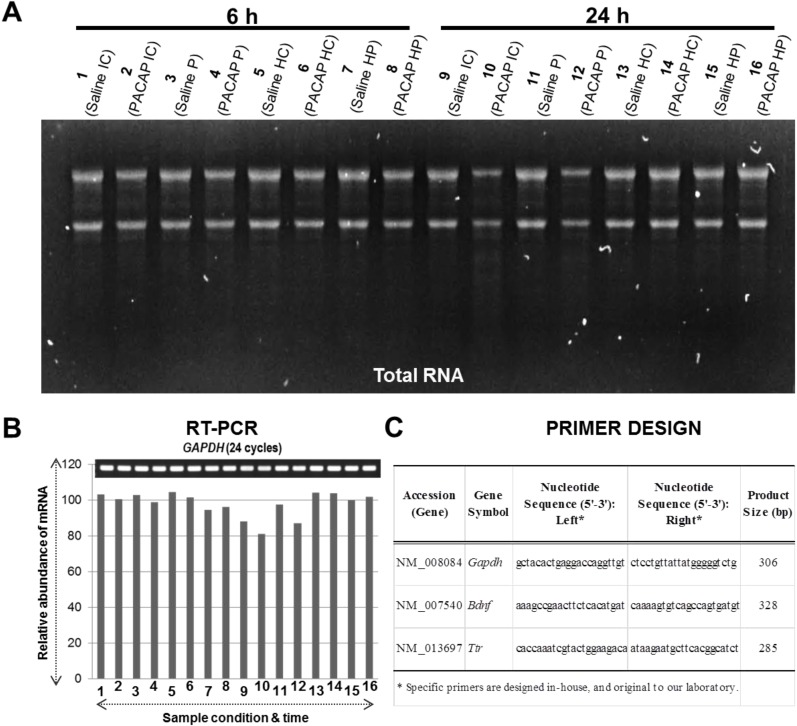
Total RNA visualized by agarose gel electrophoresis (**A**) followed by mRNA expression profiles (by RT-PCR) of *Gapdh* used a positive control (**B**) to confirm equal loading and proper cDNA synthesis. In (**A**), lane numbers 1 to 8 and 9 to 16 indicate 6 h and 24 h samples, respectively, as described. In (**B**), gel image on top show PCR product bands stained with ethidium bromide; band intensities as relative abundance of mRNA are also presented graphically below for clarity. (**C**), Primer design for genes analyzed in this study. Abbreviations: IC, ischemic core; P, penumbra; HC, healthy control; HP, healthy penumbra.

Based on our recently published preliminary study [20], we used the experimental strategy as depicted in Figure 1, where the IC and P regions were excised from the ipsilateral hemisphere post PACAP38 or saline injections, respectively. Briefly, the IC and P were demarcated based on the visual confirmation and past experience and observations post-triphenyltetrazolium chloride (TTC) staining of ischemic brains [6,12,13,14,20]. The total RNA extraction was performed as described in Materials and Methods (see below, Section 4.4.). The obtained total RNA (Figure 2A) was taken for DNA microarray analysis using two 4 × 44 K mouse whole genome oligo DNA chips (the chip, design, and samples are illustrated in Figure 1) in conjunction with a two-color, dye-swap approach ([12,13,14,22,23], and references therein). Prior to the DNA microarray analysis, the synthesized cDNA quality was confirmed by RT-PCR using an endogenous control *glyceraldehyde 3-phosphate dehydrogenase* (*Gapdh*) gene expression profile (Figure 2B). The visual image of amplified PCR bands appeared to show no major differences among the sample conditions on *Gapdh* mRNA abundance. However, using the image analysis software the calculated and graphically presented intensities for each gene expression showed a slight difference in abundance of *Gapdh* mRNA expression in some samples, which can be correlated to the obtained total RNA in Figure 2A. Nonetheless, with these gel-based analyses the good quality (see also below, Section 4.4.) of total RNA correlated well with the synthesized cDNA by RT-PCR analysis of the *Gapdh* gene, and that is used as a positive control [24]. The gene-specific primer pair for *Gapdh* is shown in Figure 2C.

The reason for presenting the experimental strategy was to make the readers aware of this highly complex and expensive analysis, and to avoid mistakes. Therefore, each step from sampling to total RNA extraction protocol, quality and quantity check was carefully checked and cross-checked before proceeding to the microarray chip experiment itself, where the main objective was to identify PACAP38 influenced gene expressions specifically in IC and P regions as compared to the first two studies carried out using whole hemispheres [12,13,14]. Our most recent data on both selected gene expressions by RT-PCR and the CRMP2 protein by Western blotting had also indicated that expression patterns for these investigated molecules varied in different brain regions following PACAP38 treatment compared to healthy contralateral regions including saline treatment [20].

### 2.2. Overview of the Ischemic Core (IC) and Penumbra (P) Differentially Expressed Genes upon PACAP38 Treatment

**Figure 3:**
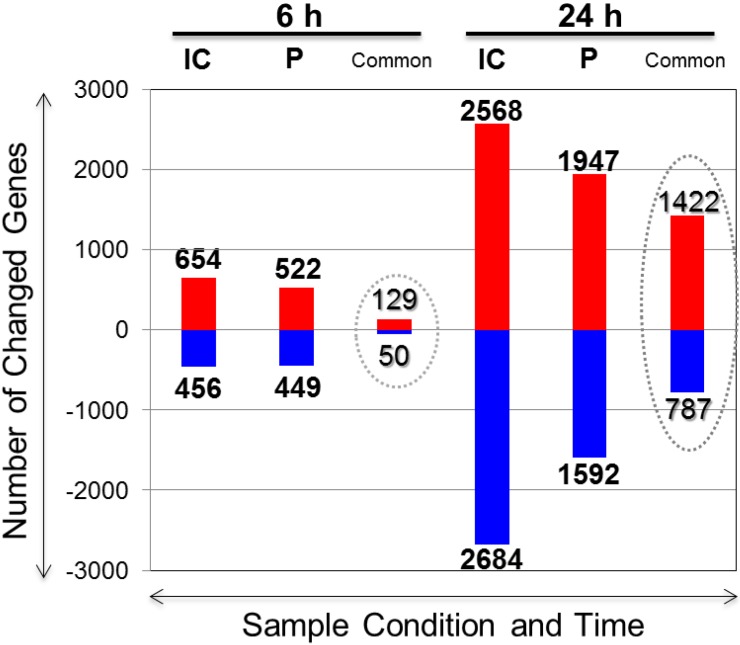
Differentially expressed PACAP38 influenced genes in the IC and P regions. The numbers above each bar (up- and down-regulated) indicate the selection of genes from the total microarray datasets within a defined fold range of greater than 1.5-fold and less than 0.75-fold. The gene lists are presented in Supplementary Tables 1–8 for the time periods 6 h and 24 h, and for each IC and P region, respectively; common genes are listed in Supplementary Tables 9–12.

Genome-wide differential gene expression profiles were obtained for IC and P regions post-treatment with PACAP38. These are graphically presented in Figure 3. A total of 654 and 456, and 522 and 449 genes were up-regulated and down-regulated in the IC and P, respectively, at 6 h; 129 (up-regulated) and 50 (down-regulated) genes were commonly expressed (Figure 3, 6 h). These gene lists are presented in the same series as above in Supplementary Tables 1–4. At 24 h, a much larger number of genes were expressed. In the IC, a total of 2568 and 2684 and 1947 and 1592 genes were up-regulated and down-regulated in the IC and P, respectively; correspondingly, 1422 (up-regulated) and 787 (down-regulated) genes were commonly expressed (Figure 3, 24 h). These gene lists are presented in the same series as above in Supplementary Tables 5–8. The common gene lists are shown in Supplementary Tables 9 (IC and P common up-regulated, 6 h), 10 (IC and P common down-regulated, 6 h), 11 (IC and P common up-regulated, 24 h), and 12 (IC and P common down-regulated, 24 h). The large-scale transcriptomic data presented here is available under the accession/series numbers GSE 62884 at the National Center for Biotechnology Information (NCBI) Gene Expression Omnibus (GEO) public functional genomics data repository [25]. Readers are referred to these gene expression profiles for further information, download, and/or independent analysis.

Looking at the time course pattern of gene expressions, as expected a greater number of genes were up- or down-regulated at 24 h compared to 6 h. Moreover, the gene expressions in the IC were greater than in the P, and more so at 24 h than over the 6 h time period. Although we identified commonly occurring genes between the IC and P regions, it is quite obvious that at 6 h, the unique genes to each region greatly outnumber the common genes, whereas as the time progresses along with ischemia, the common genes increase to a far greater number compared to that seen at 6 h. This indicates two things: first, the PACAP38 effect is initiated quite early, beginning at 6 h, where the IC is not distinct (not visually discernable) from the P region, and PACAP38, if neuroprotective as is claimed, should affect more genes that are in common to both these regions as we know that PACAP38 causes a reduction in the infarct over the saline treatment at 24 h; second, at 24 h, along with the PACAP38 influenced genes we can also see numerous gene expressions related to the ischemic response as the ischemia progresses itself with time. This is one reason behind the presence of a large number of commonly expressed genes at 24 h. Looking at the commonly expressed genes in Supplementary Tables 9 and 10 (6 h), we can see that the fold-change is not drastically different between IC and P, but at 24 h (Supplementary Tables 11 and 12), the fold-change for similar genes is quite distinct; *i.e.*, in the IC region the genes are more highly expressed (fold-change) than in the P region. For example, the interleukin 6 (*Il6*) gene, which serves as a marker for ischemia [6], is very highly expressed in the IC but not in the P region. These identified genes thus can be considered as specifically regulated by PACAP38 treatment in these two distinct regions in the ischemic hemisphere and are targets for further analysis using bioinformatics tools. Another important consideration in the current experimental strategy and subsequent DNA microarray analysis is the possibility of finding genes that could have been masked in the previous studies using whole brain/hemispheres [12,13,14], and that is exactly what we find and discuss below.

### 2.3. Identification of Two PACAP38 Induced Genes, Brain-Derived Neurotrophic Factor (Bdnf) and Transthyretin (Ttr) in the Ischemic Core (IC) and Penumbra (P) and Their Validation by RT-PCR as Examples

**Figure 4:**
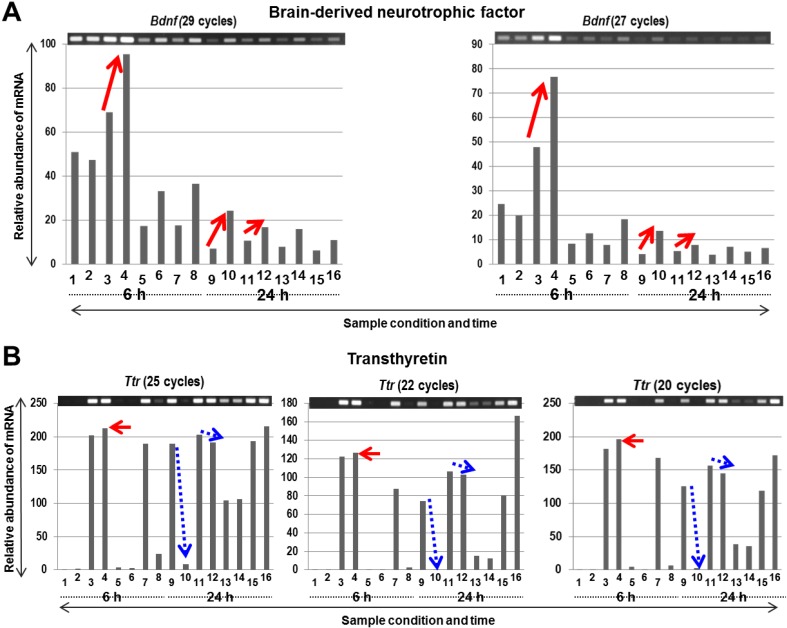
The mRNA expression profiles (by RT-PCR) of brain-derived neurotrophic factor (*Bdnf*; **A**) and transthyretin (*Ttr*; **B**) genes. The PCR product bands stained with ethidium bromide are also quantified and the relative abundance is presented below graphically. The lane numbers are the same as in Figure 2.

Among the numerous differentially expressed genes (Supplementary Tables 1–8), we were surprised to find that PACAP38 induced the expression of two known genes playing a role in neuroprotection in the brain, namely brain-derived neurotrophic factor (*Bdnf*) [26,27,28] and transthyretin (*Ttr*) [29,30,31]. Although these genes are induced to slightly elevated levels by microarray analysis, they were not identified in our previous whole hemisphere experiments [12,13,14], again reinforcing the importance of selecting/sampling these two brain regions (IC and P) in the present study. Therefore, rather than randomly selecting genes for confirmatory RT-PCR experiments, we decided to examine the expression of *Bdnf* and *Ttr* genes using gene-specific primers (provided in Figure 2C). RT-PCR analysis in all the tested samples revealed that at 6 h post-treatment with PACAP38, their (*Bdnf* and *Ttr*) mRNA abundance increased over the saline control (Figure 4A,B). Interestingly, their inductions were clearly seen in the P region at 6 h over down-regulation (*Bdnf*) or no change in expression (*Ttr*) in the IC region. In the case of *Bdnf*, at 24 h, in both the IC and the P regions, PACAP38 increased its abundance, suggesting that the *Bdnf* gene was activated, which appears to be a positive development in the recovery of the ischemia or plays a role in neuroprotection. Most recently, it was reported that endogenous injury-associated BDNF expression is critically involved in induction, but not maintenance, of injury-associated PACAP expression in sensory and motor neurons [28]. Other than this recent work, it had also previously been reported that PACAP38 induced multimodal neuroprotection in oxygen-glucose deprivation and MCAO stroke models that involved a temporary association with increased expression of BDNF and associated molecules [32].

In the case of *TTR*, similar to the microarray data, the 6 h RT-PCR results were validated, in three consecutive analyses, however, at 24 h, an opposite result was obtained, *i.e.*, a slight decrease in mRNA abundance. At present, we are unable to explain this discrepancy, although we have not tried any different primer combinations or examined the probes on the DNA chip for this gene more in depth. From the published literature, we found one study on cerebrospinal fluid (CSF), where the authors suggested that in conditions of a compromised heat-shock response, CSF TTR contributed to control neuronal cell death, edema, and inflammation, thereby influencing the survival of endangered neurons in cerebral ischemia [29]. A just published paper also provides some new hints to the neuroprotective role of TTR, namely that of a transcription inducer of insulin-like growth factor receptor 1 (IGF-IR) in the central nervous systems [31]. Nevertheless, it is emphasized that RT-PCR analysis should be diligently performed, especially for genes of interest, even for those gene candidates not showing high-fold changed values from the microarray experiment. Taken together, our present results unravel the expression of two important molecular factors involved in neuroprotection and could be almost validated independently by RT-PCR, an essential part of the microarray analysis.

### 2.4. Functional Categorization/Biological Function of the Differentially Expressed Genes by PACAP38 Treatment in Ischemic Core (IC) and Penumbra (P) Regions Reveals Activation of Neuroprotective Mechanisms

As part of a bioinformatics approach to examine the functions of these changed gene expressions, we utilized the pathway- and disease state-focused gene classifications available on the QIAGEN website (SABiosciences; www.sabiosciences.com) to reveal the trend of predominant pathways affected in the IC (Supplementary Figures 1 and 3, 6 and 24 h, respectively) and P (Supplementary Figures 2 and 4, 6 and 24 h, respectively) regions. The PACAP38 up- and down-regulated genes at 6 h and 24 h were classified based on the available categories of more than 100 biological pathways. This categorization revealed that the trends of gene quantity (numbers) and quality (function) in the respective pathways varied between the two brain regions IC and P. Here we primarily focus on and discuss some of these up-regulated gene functions to explain in part the possible mechanisms behind PACAP38-induced neuroprotection in the brain in the context of our experimental approach.

In the case of the IC post-6 h PACAP38 treatment, the top categories of molecular toxicology pathway finder, mesenchymal stem cell, innate and adaptive immune response, inflammatory responses, and autoimmunity showed more up-regulated genes compared to the down-regulated genes. In contrast, in the P post-6 h PACAP38 treatment, except for mesenchymal stem cell function-related genes that were up-regulated as a top 4th category, these other functions predominant in IC were not present in the top list of up-regulated functions. This suggests that PACAP38 influences molecules early on in the IC rather than the P regions, and these are predominantly involved with the immune and inflammatory responses. However, when we look at the top up-regulated gene lists in IC (Supplementary Table 1) and P (Supplementary Table 3), we find that as a matter of fact the top genes in both regions are related to the immune response; *i.e.*, functional categorization is more of a quantitative listing of genes rather than qualitative.

For example, in the case of the IC, 6 h after PACAP38 treatment, the top up-regulated gene was cathelicidin antimicrobial peptide (*Camp*), which exhibits both antibacterial activity and function as chemoattractant for immune cells [33]. Though we do not know much about this protein function in the innate immune system in the brain, a recent study in the rat revealed that rCRAMP has a role in brain immunity via the stimulation of cytokine production and glial cell activation and protecting brain cells by inducing neurotrophic factors [34]. Is there a similar function for *Camp* in the infarct core? We have yet to answer this question. Another novel gene identified here was the alpha hemoglobin stabilizing protein (*Ahsp*) [35], which was initially identified as an erythroid-specific chaperon protein that as the name indicates specifically stabilizes the α-subunit of hemoglobin [36]. To note, the *Ahsp* gene expression was the second highest fold change observed only in the IC at 6 h (Supplementary Table 1). This is quite interesting as it has been reported that AHSP neutralizes harmful effects of free α-hemoglobin in not only normal erythropoiesis but also under disease [36,37]. It was also revealed thereafter that interaction between AHSP and α-hemoglobin made the latter non-reactive preventing oxidative damage *in vivo* [38]. Most recently, and relevant to this study, was the discovery that *Ahsp* gene expression increased in K562 cells upon cytokine *Il6*-induced STAT3 activation [39]. As discussed below in this section, the *Il6* gene expression, which is highly up-regulated at 24 h (but only weakly at 6 h; Supplementary Table 1), might be acting as a neuroprotective factor under the PACAP38 treatment. Is *Ahsp* expression, therefore, related to the prevention of a pro-oxidant activity of α-hemoglobin, which might be released in the infarct core? At present we have no data to support our view but it would be interesting to look at the role of both *Ahsp* and α-hemoglobin in the ischemic brain with or without PACAP38 treatment.

In the case of the P, 6 h after PACAP38 treatment, the top up-regulated gene encoded an interferon beta 1 (fibroblast) protein, *Ifnb1* [40]. Again, as for the *Camp* gene, the *Ifnb1* function in brain ischemia remains to be clarified. The type I IFNs are known to exert antiproliferative, proapoptotic antiangiogenic, and immunemodulatory functions, and interestingly, *Ifnb1* was very recently shown to induce autophagy in Michigan Cancer Foundation-7 (MCF-7) breast cancer cells promoting their survival [41]. In other words, IFNB1 mediated its antiproliferative effects independent of autophagy [41]. Another evidence of its anti-inflammatory and immunemodulatory functions has also come from mouse models of cerebral malaria [42], where IFNB1 treatment was shown to cause the down-regulation of pro-inflammatory cytokines subsequently increasing overall survival against malaria [43]. Using these two examples, we can say that at initial stages of ischemia with PACAP38 treatment the brain cells respond to the insult by triggering induction of molecules with protective/anti-inflammatory roles. This also indirectly through these gene inductions implies and supports a neuroprotective role for PACAP38. Although we have not examined in detail the mesenchymal stem cell gene functions, there are some reports on the therapeutic benefits of bone marrow stromal cell injections following cerebral ischemia in the rat [44] and autologous mesenchymal stem cell transplantation in patients with ischemic stroke [45]. A recent study has also reported the use of mesenchymal stem cells as a promising tool for infarcted myocardia and strokes due to their ability to promote endogenous angiogenesis and neurogenesis via diverse secreted factors [46]. These will be investigated in future studies.

At 24 h, some different gene function lists are evident as the top categories in both the IC and P regions compared to the 6 h time period. For example, in IC among the top gene functions are G protein coupled receptors and dendritic and antigen presenting cell, and those are common to both IC and P regions. These data suggest that at the 24 h time period ischemic response is more pronounced and there are more similarities between the IC and P region gene expressions, which are also suggested by the number of commonly expressed genes seen in Figure 3. On the other hand, it does not imply that the immune/inflammatory response genes were not induced, which must be expected considering that PACAP38 treatment also reduces the infarct size [12,13,14]. Searching the gene lists we found that the *Ifnb1* gene was also strongly up-regulated in both IC and P at 24 h (Supplementary Tables 5 and 7), suggesting that beta interferon may be a candidate gene for PACAP38 induced neuroprotection. Other than this specific interferon beta gene (*Ifnb1*) identified in this study, we found that numerous chemokines, namely the chemokine (C-C motif) ligand (CCL) subfamily (*Ccl2*, *3*, *4*, *5*, *6*, *7*, *9*, *11*, *12*, and *24*) along with chemokine (C-X-C motif) ligand (CXCL) subfamily (*Cxcl1*, *2*, *3* and *10*) were predominant and highly up-regulated molecules in both IC and P regions (Supplementary Tables 5 and 7). The ischemic marker cytokine, *Il6* gene induction was also seen along with these chemokines with PACAP38 treatment. We discuss further down the *Il6*, which has dual roles as a neurodegenerative [47] and neuroprotective [48,49,50] factor.

A most recent report investigating the effect of low concentrations of the neurotoxicant methylmercury in cultured microglia demonstrated a role for astrocyte-induced (involving purinergic P2Y_1_ receptor activation followed by calcium oscillation) IL-6-mediated neuroprotection of neurons [51]. Taken together, PACAP38 neuroprotection could also be linked through activation of anti-inflammatory chemokines and the neuroprotective *Il6*, however the combination of gene events may vary between the IC and P regions. In addition, the role of astrocytes in neuroprotection can be speculated based on the fact that these cells are major producers of the CC and CXC subfamily chemokines and have a role in the regulation of central nervous system autoimmunity [52,53,54]. In the above context, we would also like to mention a recently published study dealing with the *Ccl3*, *Ccr2*, and *Cxcl10* [55]. Using mice deficient in *Ccl3*, *Ccr2* or *Cxcl10*, the interaction among chemokine signals in regulating dendritic cells in an acute brain injury model was clarified in conjunction with DNA microarray analysis. These authors demonstrated a correlation between these chemokine signals and that their interaction in the injured brain, setting the stage for inflammatory cell activation involving not only the resident microglia and astrocytes but also invading immune cells from the periphery after trauma [55]. We also speculate that a similar mechanism is in play in the IC and P regions, where chemokines are highly expressed (Supplementary Tables 5 and 7). To note, *Ccr2* gene expression is also induced by PACAP38 in both the IC and P regions, albeit at a lower-fold change compared to the chemokines themselves.

### 2.5. Biological Functions of PACAP38 Affected Genes by Ingenuity Pathway Analysis (IPA) Reveals Novel Molecular Networks Operating within the Ischemic Brain

**Figure 5:**
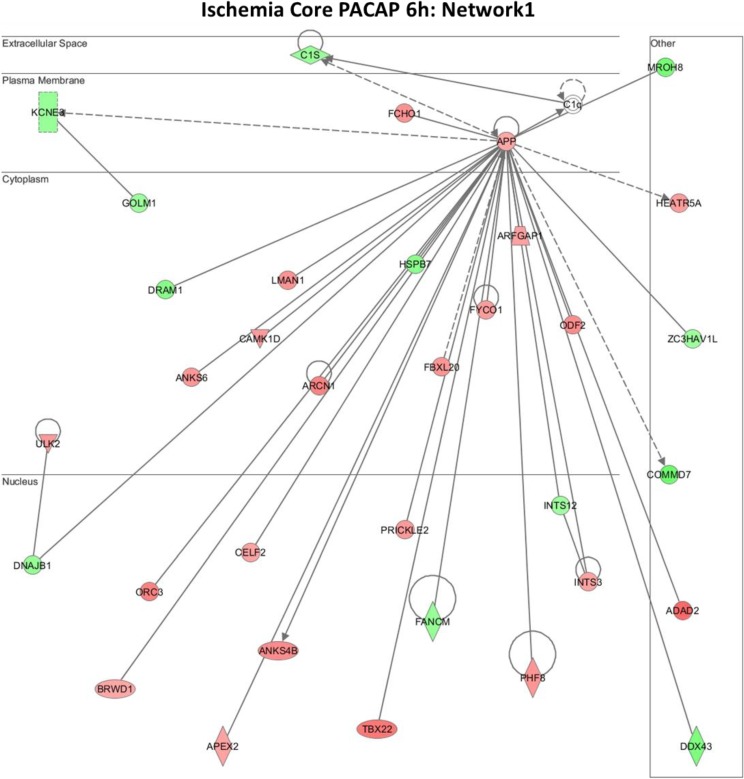
The top network 1 for the ischemic core at 6 h following PACAP38 treatment.

**Figure 6:**
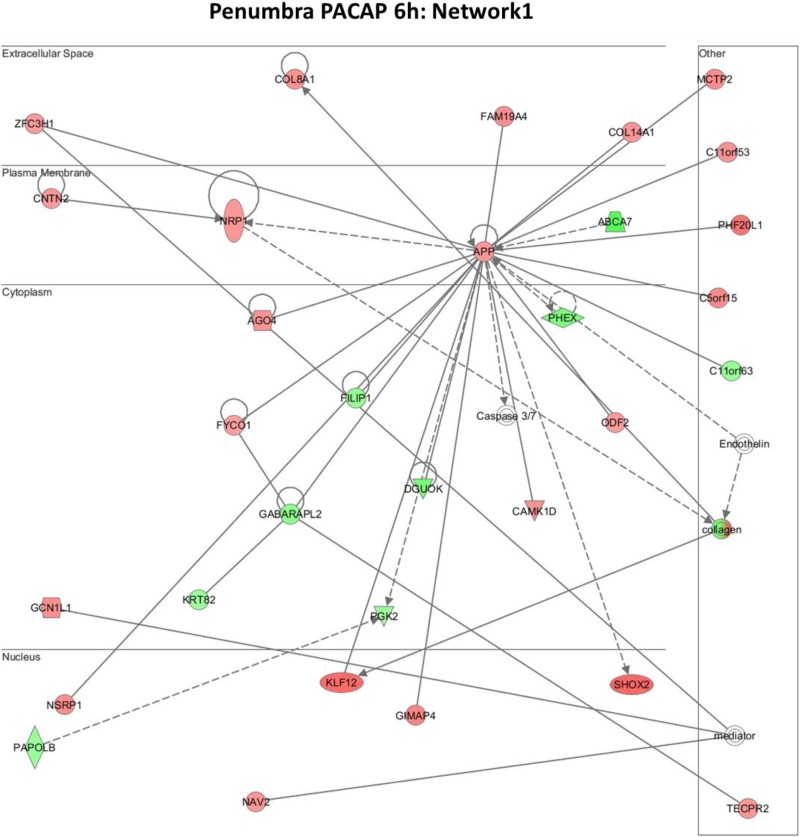
The top network 1 for the penumbra at 6 h following PACAP38 treatment.

Further functional annotation of the differentially expressed genes was carried out using the IPA platform, where networks are generated on the basis of known functions and interconnectivity of affected genes. Multiple networks were generated as above for both the IC and P regions at 6 and 24 h, considering the large volume of data and our interest in the early changes or influence of PACAP38, we select and present the results of the top network 1 at the 6 h time point in the present study. The reason behind discussing only the first networks is that there is a vast amount of data from both up-regulated and down-regulated gene lists in both the brain regions. Their analyses will be a detailed process and the analyzed outputs will be the subject of a new paper. The networks 1 for IC and P regions are shown in Figure 5 (IC) and Figure 6 (P), respectively. The molecules involved in these networks are listed in Table 1 (IC) and Table 2 (P).

**Table 1:** Molecules for Network 1, PACAP 6 h for the Ischemic core (IC).

Symbol	Entrez Gene Name	Agilent	Fold Change	Networks	Location	Type(s)
COMMD7	COMM domain containing 7	A_51_P459550	−2.27	1	Other	other
MROH8	maestro heat-like repeat family member 8	A_52_P263962	−2.13	1	Other	other
DDX43	DEAD (Asp-Glu-Ala-Asp) box polypeptide 43	A_52_P98287	−2.00	1	Other	enzyme
DRAM1	DNA-damage regulated autophagy modulator 1	A_51_P481482	−1.85	1	Cytoplasm	other
HSPB7	heat shock 27kDa protein family, member 7 (cardiovascular)	A_51_P346445	−1.79	1	Cytoplasm	other
DNAJB1	DnaJ (Hsp40) homolog, subfamily B, member 1	A_51_P153486	−1.61	1	Nucleus	other
FANCM	Fanconi anemia, complementation group M	A_51_P476030	−1.61	1	Nucleus	enzyme
C1S	complement component 1, s subcomponent	A_52_P39505	−1.59	1	Extracellular Space	peptidase
KCNE3	potassium voltage-gated channel, Isk-related family, member 3	A_51_P336599	−1.59	1	Plasma Membrane	ion channel
GOLM1	golgi membrane protein 1	A_51_P171200	−1.52	1	Cytoplasm	other
ZC3HAV1L	zinc finger CCCH-type, antiviral 1-like	A_52_P260747	−1.52	1	Other	other
INTS12	integrator complex subunit 12	A_52_P1155474	−1.47	1	Nucleus	other
APP	amyloid beta (A4) precursor protein	A_52_P381311	1.44	1	Plasma Membrane	other
BRWD1	bromodomain and WD repeat domain containing 1	A_52_P298237	1.44	1	Nucleus	transcription regulator
APEX2	APEX nuclease (apurinic/apyrimidinic endonuclease) 2	A_52_P417148	1.50	1	Nucleus	enzyme
INTS3	integrator complex subunit 3	A_52_P228684	1.50	1	Nucleus	other
ARFGAP1	ADP-ribosylation factor GTPase activating protein 1	A_52_P207361	1.54	1	Cytoplasm	transporter
FYCO1	FYVE and coiled-coil domain containing 1	A_52_P265666	1.59	1	Cytoplasm	other
HEATR5A	HEAT repeat containing 5A	A_52_P601569	1.59	1	Other	other
CAMK1D	calcium/calmodulin-dependent protein kinase ID	A_52_P804224	1.63	1	Cytoplasm	kinase
PRICKLE2	prickle homolog 2 (Drosophila)	A_52_P201482	1.63	1	Nucleus	other
ULK2	unc-51 like autophagy activating kinase 2	A_52_P226137	1.63	1	Cytoplasm	kinase
ANKS6	ankyrin repeat and sterile alpha motif domain containing 6	A_51_P298933	1.69	1	Cytoplasm	other
FBXL20	F-box and leucine-rich repeat protein 20	A_52_P35477	1.72	1	Cytoplasm	other
PHF8	PHD finger protein 8	A_51_P117369	1.72	1	Nucleus	enzyme
FCHO1	FCH domain only 1	A_51_P228777	1.77	1	Plasma Membrane	other
LMAN1	lectin, mannose-binding, 1	A_51_P264984	1.77	1	Cytoplasm	other
ODF2	outer dense fiber of sperm tails 2	A_51_P113162	1.85	1	Cytoplasm	other
ANKS4B	ankyrin repeat and sterile alpha motif domain containing 4B	A_51_P318618	1.86	1	Nucleus	transcription regulator
ARCN1	archain 1	A_52_P1004491	2.03	1	Cytoplasm	other
ORC3	origin recognition complex, subunit 3	A_52_P577438	2.04	1	Nucleus	other
TBX22	T-box 22	A_52_P39481	2.19	1	Nucleus	transcription regulator
ADAD2	adenosine deaminase domain containing 2	A_51_P140042	2.43	1	Other	other
C1q	--	--		1	Plasma Membrane	complex

Genes highlighted in colored boxes are similar between IC and P in Table 1 and Table 2.

**Table 2:** Molecules for Network 1, PACAP 6 h for the Penumbra (P).

Symbol	Entrez Gene Name	Agilent	Fold Change	Networks	Location	Type(s)
ABCA7	ATP-binding cassette, sub-family A (ABC1), member 7	A_52_P4928	−2.56	1	Plasma Membrane	transporter
DGUOK	deoxyguanosine kinase	A_51_P385237	−2.08	1	Cytoplasm	kinase
PHEX	phosphate regulating endopeptidase homolog, X-linked	A_51_P468249	−2.04	1	Cytoplasm	peptidase
GABARAPL2	GABA(A) receptor-associated protein-like 2	A_52_P521475	−1.75	1	Cytoplasm	other
FILIP1	filamin A interacting protein 1	A_51_P333438	−1.67	1	Cytoplasm	other
C11orf63	chromosome 11 open reading frame 63	A_51_P258473	−1.61	1	Other	other
PAPOLB	poly(A) polymerase beta (testis specific)	A_51_P310333	−1.59	1	Nucleus	enzyme
KRT82	keratin 82	A_51_P239367	−1.54	1	Cytoplasm	other
PGK2	phosphoglycerate kinase 2 , PGK-2	A_51_P125487	−1.52	1	Cytoplasm	kinase
FYCO1	FYVE and coiled-coil domain containing 1	A_52_P265666	1.44	1	Cytoplasm	other
ODF2	outer dense fiber of sperm tails 2	A_51_P113162	1.49	1	Cytoplasm	other
ZFC3H1	zinc finger, C3H1-type containing	A_52_P122393	1.50	1	Extracellular Space	other
CNTN2	contactin 2 (axonal)	A_52_P651870	1.52	1	Plasma Membrane	other
APP	amyloid beta (A4) precursor protein	A_52_P110982	1.55	1	Plasma Membrane	other
NRP1	neuropilin 1	A_51_P469285	1.55	1	Plasma Membrane	transmembrane receptor
TECPR2	tectonin beta-propeller repeat containing 2	A_51_P130282	1.56	1	Other	other
NAV2	neuron navigator 2	A_52_P551829	1.57	1	Nucleus	other
COL14A1	collagen, type XIV, alpha 1	A_51_P141467	1.59	1	Extracellular Space	other
AGO4	argonaute RISC catalytic component 4	A_52_P17422	1.61	1	Cytoplasm	translation regulator
C11orf53	chromosome 11 open reading frame 53	A_51_P173107	1.62	1	Other	other
CAMK1D	calcium/calmodulin-dependent protein kinase ID	A_52_P804224	1.65	1	Cytoplasm	kinase
COL8A1	collagen, type VIII, alpha 1	A_52_P282058	1.65	1	Extracellular Space	other
GCN1L1	GCN1 general control of amino-acid synthesis 1-like 1 (yeast)	A_52_P285100	1.67	1	Cytoplasm	translation regulator
FAM19A4	family with sequence similarity 19 (chemokine (C-C motif)-like), member A4	A_52_P54770	1.69	1	Extracellular Space	other
MCTP2	multiple C2 domains, transmembrane 2	A_51_P154913	1.70	1	Other	other
NSRP1 (=Ccdc55)	nuclear speckle splicing regulatory protein 1	A_52_P172798	1.71	1	Nucleus	other
C5orf15	chromosome 5 open reading frame 15	A_52_P33041	1.78	1	Other	other
GIMAP4	GTPase, IMAP family member 4	A_52_P205001	1.83	1	Nucleus	other
PHF20L1	PHD finger protein 20-like 1	A_52_P180283	2.10	1	Other	other
KLF12	Kruppel-like factor 12	A_51_P118877	2.23	1	Nucleus	transcription regulator
SHOX2	short stature homeobox 2	A_52_P356698	2.28	1	Nucleus	transcription regulator
Caspase 3/7	--	--		1	Cytoplasm	group
collagen	--	--		1	Other	group
Endothelin	--	--		1	Other	group
mediator	--	--		1	Other	complex

Genes highlighted in colored boxes are similar between IC and P in Table 1 and Table 2.

Immediately, and at first glance, we see that the amyloid precursor protein (APP) gene is the central molecule at the plasma membrane in both IC and P regions (Figure 5 and Figure 6). The APP has been implicated in numerous biological processes such as cell adhesion, gene transcription, neuronal differentiation, migration, neurite outgrowth, and synapse formation [56]. This protein has most recently been demonstrated to play a role in the GABAergic system for controlling adult hippocampal neurogenesis, and those authors suggest that APP dysfunction contributes to impaired neurogenesis and cognitive decline associated with Alzheimer’s disease (AD) [57]. Despite having no experimental evidence, our IPA data revealing the *APP* gene as a focal point indicate towards its (APP) role in PACAP38 mediated neuroprotection, a potentially new finding. Other than the *APP* gene, a few other molecules were found to share the network 1, as indicated in Table 1 and Table 2 (highlighted). These examples from the functionally characterized genes (Supplementary Figures 1–4) and inventory of gene lists (Supplementary Tables 1–8) that have been discussed in this section and the preceding Section 2.4, provide some insight into how PACAP38 treatment regulates the gene functions in the ischemic brain regions IC and P.

As our interest was also on the differences between the IC and P between these networks, we will discuss those molecules, in particular to make sense of the fact the P region plays or might play a much bigger role in reducing the IC after PACAP38 treatment. Three molecules are of particular interest, namely, neuropilin 1(*NRP1*) [58], tectonin beta-propeller repeat containing 2 (*TECPR2*) [59], and neuron navigator 2 (*NAV2*) [60]. The NRP1 is a multipurpose molecule that has a role in angiogenesis, axon guidance, cell survival, migration, and invasion. The TECPR2 has a role in the autophagic pathway, and its dysfunction may be involved in autophagy-associated neurodegenerative diseases. The NAV2 is a cytoskeletal-interacting protein that functions in neurite outgrowth and axonal elongation. Although further detailed bioinformatics and functional analysis will be needed to unravel the potential mechanisms behind PACAP38 neuroprotection of the ischemic brain, our targeted DNA microarray approach has put forth new data and information on PACAP38 effects in the IC and P regions of the ischemic brain. We hope that the researchers will use the publically available data at the NCBI, GEO site (under accession number GSE62884) [25] for in-depth analysis, and we welcome any new collaboration in this direction.

## 3. Concluding Remarks

The experimental strategy designed and used in this present study, namely genome-wide transcriptome profiling of specifically the IC and P brain regions after treatment with PACAP38, has helped provide an inventory of genes influenced by PACAP38 in these regions complementing those gene lists obtained with the whole hemisphere in previous studies [12,13,14]. Here we would like to emphasize that this current study utilized a small set of samples (*i.e.*, brain regions from three and two mice each in the PACAP38-treated groups 3 and 4, respectively), which can be considered a limitation of our study. However, as the aim was a DNA microarray analysis, we utilized a dye-swap approach to provide a confident gene selection in this study, which undoubtedly will have to be verified in further studies by not only our group but also other groups. In this context, the gene expression data resource is deposited at the NCBI GEO public functional genomics data repository with the series number GSE 62884 [25] and is freely accessible to interested researchers for further study and/or analyses. Although we have obtained a vast amount of data in this and previous studies, it is now the time to do (i) further meta-analyses of ischemic-related and PACAP38-influenced transcripts in the mouse brain hemisphere and regions (IC and P) to identify mechanisms of action with a bigger goal towards designing therapies for stroke/brain injury; this will also involve co-analyzing data obtained from other stroke models and treatments, and (ii) identification and analysis of numerous PACAP38-target transcripts that are unknown (not annotated or hypothetical) at present and linking them with the known (annotated) biological function transcripts for deeper insight into the signal pathways that operate in the ischemic brain alone and post-PACAP38 treatment. As kindly suggested by the anonymous reviewer, it would be worthy looking at the differential gene expressions in the IC and P regions minus the PACAP38 treatment to specifically know the progression of the infarct core. Finally, these new bioinformatics and functional genomics studies will be the only way forward to understand how and why PACAP38 is neuroprotective in the brain for any meaningful development of a stroke therapy using PACAP38 or its analogs.

## 4. Materials and Methods

### 4.1. Animals and Husbandry

Animal care and experimental procedures were used as approved by the Institutional Animal Care and Use Committee of Showa University (School of Medicine), Tokyo, Japan. Twenty-four male mice (C57BL/6J) 9 weeks of age (25~35 g body weight) were purchased from Charles River (Kanagawa, Japan). Mice were housed at the Animal Institution in Showa University in acrylic cages (eight mice/cage) maintained at 23 °C with a standard 12 h light/dark cycle, optimum humidity, and temperature control. Animals were given access to tap water and laboratory chow *ad libitum*.

### 4.2. Permanent Middle Cerebral Artery Occlusion (PMCAO), PACAP38 and Saline Treatments

The experimental design is presented in Figure 1. The PMCAO model mice were generated as described previously [12,13]. Briefly, mice were first anesthetized with 4% sevoflurane (induction) and 2% sevoflurane (maintenance) in a 30% oxygen (O_2_) and 70% nitrous oxide (N_2_O) gas mixture via a face mask, followed by an incision in the cervical skin, opening of salivary gland, resulting in visualization of the right common carotid artery. A midline cervical incision was made to expose the external carotid artery, and using intraluminal filament technique the PMCAO model was generated. PACAP38 (1 µL containing 1 pmol) or 1 µL of saline (0.9% sodium chloride (NaCl), as control) was injected i.c.v. immediately after PMCAO. PACAP38 (Peptide Institute Inc., Osaka, Japan; supplier temperature was −20 °C) was dissolved at 10^−5^ M concentration by saline, and stored at −80 °C. PACAP test solution (for injection) was diluted ×10 times with 0.9% NaCl just before use. After injections, the animals were returned to their cages. A total of four groups were prepared and used based on neurological grades (NG) as described previously [12,13,14,20]. These are Group 1: PMCAO injected normal saline and decapitated after 6 h (eight animals); Group 2: PMCAO injected normal saline and decapitated after 24 h (10 animals); Group 3: PMCAO injected PACAP38 and decapitated after 6 h (three animals); and Group 4: PMCAO injected PACAP38 and decapitated after 24 h (three animals). For three animals each from Groups 1 to 3 and two animals in Group 4 with scores NG1 and NG2 were used for dissecting the brains and sampling the IC and P regions for downstream microarray analysis. Animals not used in this study were as follows: Group 1, two animals showing NG0 and one animal NG3; Group 2, two animals showing NG0 and three animals NG3; Group 4, one animal showing NG3.

### 4.3. Dissection of Brain, Sampling, and Storage

Six or twenty-four hours post-injection of PACAP38 or saline, the mice were removed from their cages, decapitated, and their brains carefully removed on ice. The right (ipsilateral; ischemic) and left (contralateral) hemispheres were dissected, and from each hemisphere the IC and P regions and corresponding healthy control (HC) and healthy penumbra (HP) were carefully removed with a sterile scalpel and placed in 2 mL Eppendorf microfuge tubes. The samples were then quickly immersed in liquid nitrogen and stored in −80 °C prior to further analysis (Figure 1).

### 4.4. Total RNA Extraction, Synthesis of cDNA, and Reverse Transcription-Polymerase Chain Reaction

Stored brain regions were ground to a very fine powder with liquid nitrogen, and the total RNA was extracted the quantity and quality of which was determined and confirmed exactly as described previously [12,13,14,20]. Individual samples were pooled for each condition (control and treatment) as the amount of each region was very small, and the resulting powder was used for total RNA extraction. Briefly, the total RNA was extracted using an optimized protocol based on the QIAGEN RNeasy Mini Kit (QIAGEN, Germantown, MD, USA). We measured the obtained total RNA spectrophotometrically with NanoDrop (Thermo Scientific, Wilmington, DE, USA), which showed good quality (A_260/280_ > 1.8; A_260/230_ > 1.8) total RNA was obtained in optimum quantity (>300 ng/µL), followed by band visualization using formaldehyde-agarose gel electrophoresis further confirming no visible damage and degradation (Figure 2A). For validation of total RNA quality and subsequently synthesized cDNA, RT-PCR was carried out using a commonly used house-keeping gene *Gapdh* as positive control [12,13,14,20,24]. The gene-specific primers were designed (Figure 2C). The cDNA synthesis and RT-PCR analysis protocol used is as follows: Total RNA samples were first DNase-treated with RNase-free DNase (Stratagene, Agilent Technologies, La Jolla, CA, USA). First-strand cDNA was then synthesized in a 20 μL reaction mixture with an AffinityScript QPCR cDNA Synthesis Kit (Stratagene) according to the protocol provided by the manufacturer, using 1 μg total RNA. The reaction conditions were 25 °C for 5 min, 42 °C for 5 min, 55 °C for 40 min and 95 °C for 5 min. The synthesized cDNA was made up to a volume of 50 µL with sterile water supplied in the kit. The reaction mixture contained 0.6 μL of the first-strand cDNA, 7 pmols of each primer set and 6.0 µL of the Emerald Amp PCR Master Mix (2X premix) (TaKaRa Shuzo, Otsu, Shiga, Japan) in a total volume of 12 µL. Thermal cycling (Applied Biosystems, Tokyo, Japan) parameters were as follows: after an initial denaturation at 97 °C for 5 min, samples were subjected to a cycling regime of 20 to 40 cycles at 95 °C for 45 s, 55 °C for 45 s, and 72 °C for 1 min. At the end of the final cycle, an additional extension step was carried out for 10 min at 72 °C. After completion of the PCR the total reaction mixture was spun down and mixed (3 µL), before being loaded into the wells of a 1.2% or 1.8% agarose (Agarose (fine powder) Cat no. 02468-95, Nacalai Tesque, Kyoto, Japan) gel. Electrophoresis was then performed for ~22 min at 100 Volts in 1X TAE buffer using a Mupid-ex electrophoresis system (ADVANCE, Tokyo, Japan). The gels were stained (8 µL of 10 mg/mL ethidium bromide in 200 mL 1X TAE buffer) for ~7 min and the stained bands were visualized with the ChemiDoc XRS+ imaging system (Bio-Rad Laboratories, Inc., Hercules, CA, USA). Each gene expression analysis was performed at least twice or thrice as independent PCR reactions and electrophoresis on gel, and one of the images was presented as a representative data for each gene in the respective figures for up-/down-regulated expressions.

### 4.5. DNA Microarray Analysis

In order to confirm differing gene expressions between IC and P, the tissues were individually ground to a very fine powder with liquid N_2_ followed by genome-wide analysis as described previously [12,13]. Briefly, sample powders were stored at −80 °C till used for RNA or DNA extraction. Following total RNA extraction, quality and quantity check, cDNA synthesis and RT-PCR, the sample was considered to be appropriate for use in the DNA microarray analysis. The reasons for multiple checks and use prior to DNA microarray analysis was first due the high cost incurred during this analysis, and second, and most importantly, to obtain as accurate results as possible on gene expression changes that could be easily misinterpreted and used in case should any of the above steps not be followed precisely. Total RNA extracted from each tissue (IC and P, PACAP38/saline injected) experiment was pooled (250 ng) in each group prior to DNA microarray analysis (Agilent mouse whole genome 4 × 44 K; G4122F; Agilent Technologies, Santa Clara, CA, USA) performed essentially as described previously [12,13]. We performed DNA microarray analysis using two slides composed of four chips each, labeling one set (control and treatment) with Cy 3 and the other with Cy 5 for each IC and P at 6 and 24 h, and as per the Agilent instructions using the established dye-swap approach [12,13,22,23].

### 4.6. Access to Gene Array Data

The outputs of microarray analysis used in this study are available under the series number GSE 62884 [25], at the NCBI GEO public functional genomics data repository [61].

### 4.7. Functional Categorization

Pathway and disease state-focused gene classification of the differentially expressed genes in the IC and P of ischemic brain mouse model were classified based on the available categories of more than 100 biological pathways or specific disease states in the SABiosciences PCR array list (QIAGEN; www.sabiosciences.com) for *Mus musculus*. The numbers in the y-axis represent the numbers of genes in each category, which are indicated on the x-axis.

### 4.8. Ingenuity Pathway Analysis (IPA)

The biological function and network analysis were generated through the use of IPA (Ingenuity® Systems, www.ingenuity.com). The data set from microarray (6 h, IC and P, PACAP38 treatment), which are the differentially expressed (≧/≦ 1.5/0.75-fold compared to saline control) genes, and their corresponding fold change values were uploaded as an Excel spread sheet into the IPA tool. To create gene networks, genes were overlaid onto a global molecular network developed from information contained in the ingenuity knowledge base. The functional analysis identified the biological functions that were most significant to the data set (*p*-value < 0.05) according to Right-tailed Fisher’s exact test.

## Supplementary Files

Supplementary File 1
