# Supplementary material for: Unraveling the Specific Ischemic Core and Penumbra Transcriptome in the Permanent Middle Cerebral Artery Occlusion Mouse Model Brain Treated with the Neuropeptide PACAP38
Source: Microarrays (Basel). 2015 Jan 21;4(1):2–24. doi: 10.3390/microarrays4010002 (PMC4996388; doi:10.3390/microarrays4010002)
Supplement: Supplementary File 1 [file microarrays-04-00002-s001.zip › SUPP DATA/Supplementary Figure 4.pptx]

## Slide 1
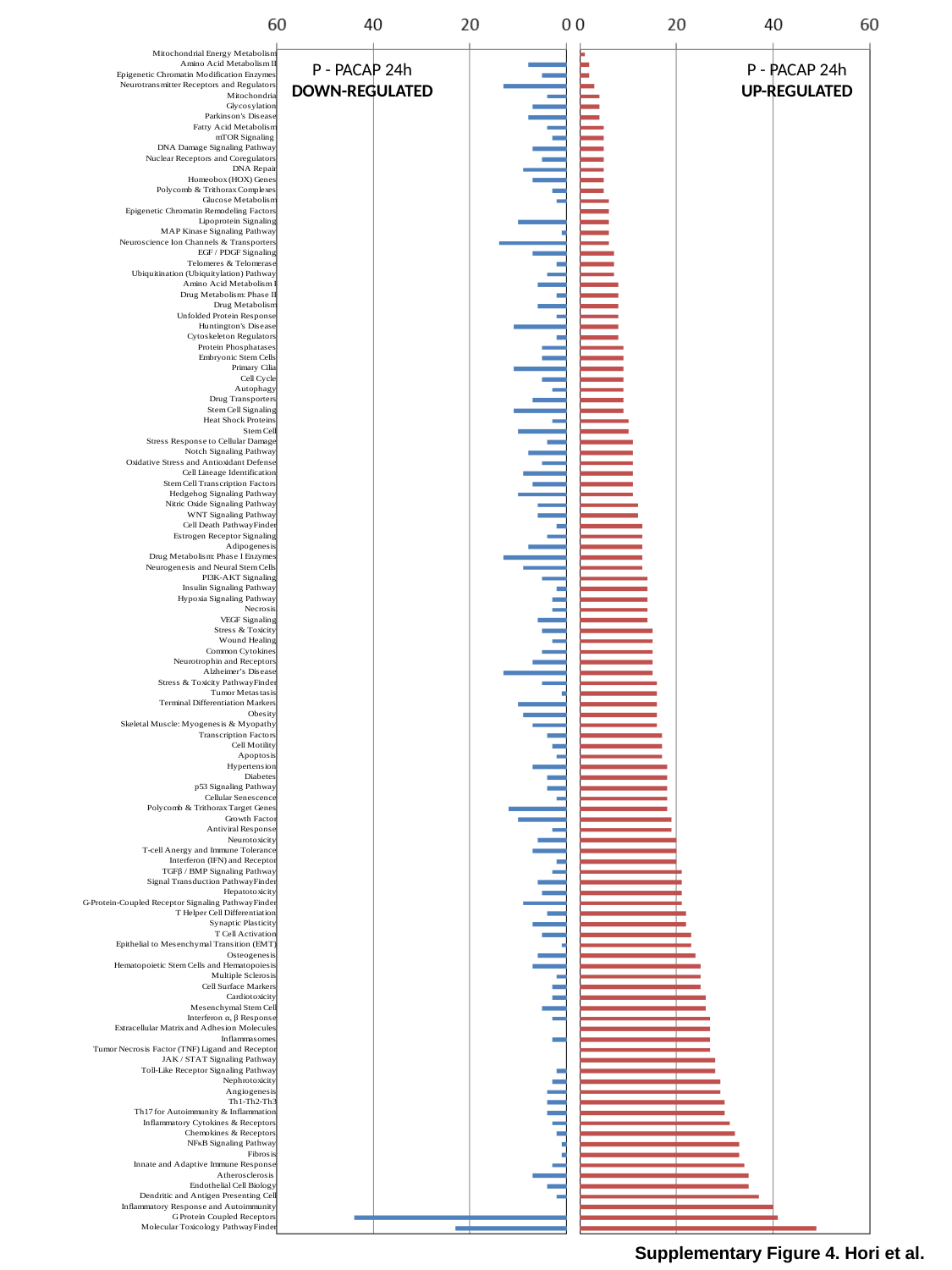

P - PACAP 24h
DOWN-REGULATED
P - PACAP 24h
UP-REGULATED
Supplementary Figure 4. Hori et al.

## Slide 2
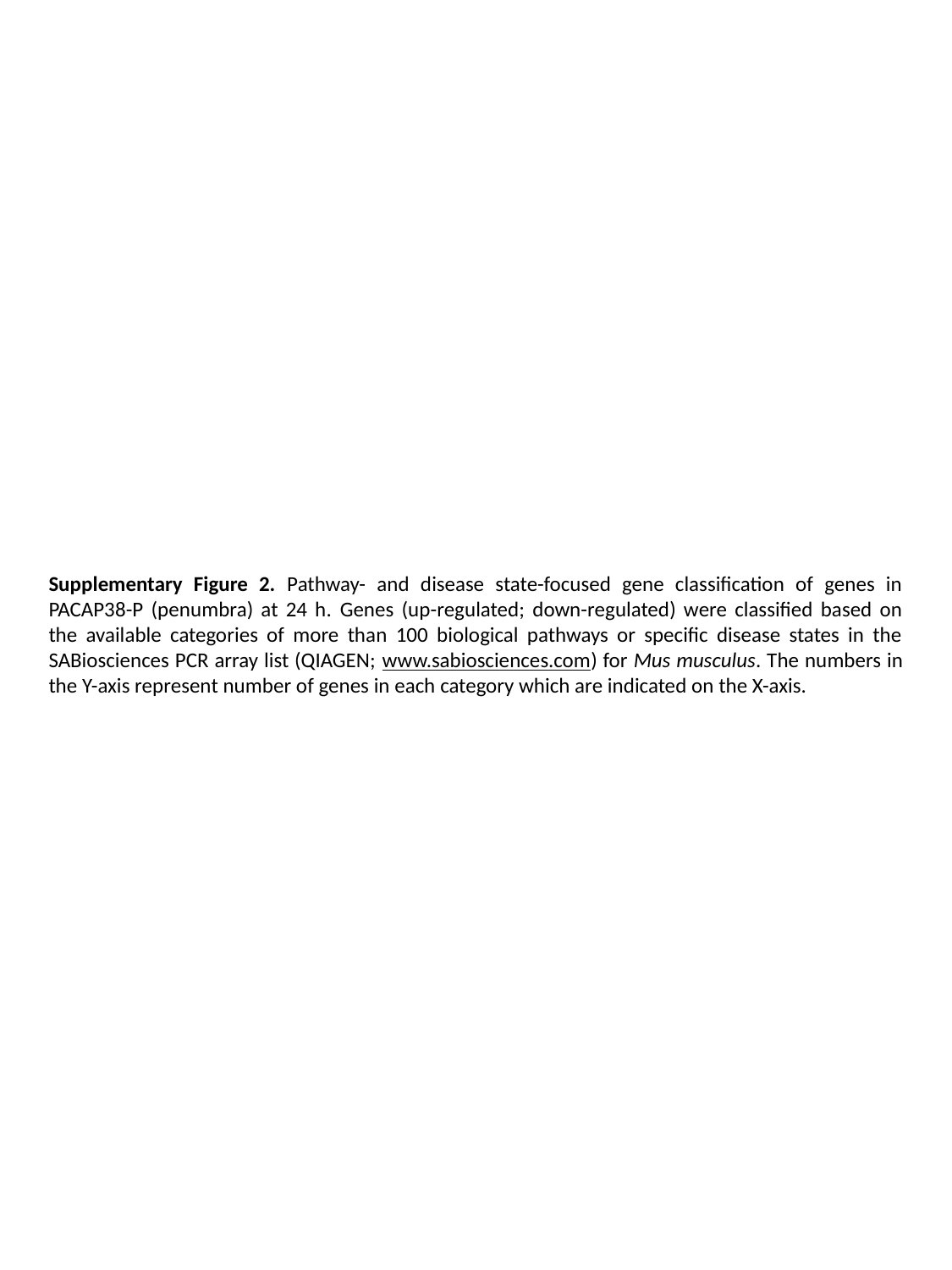

Supplementary Figure 2. Pathway- and disease state-focused gene classification of genes in PACAP38-P (penumbra) at 24 h. Genes (up-regulated; down-regulated) were classified based on the available categories of more than 100 biological pathways or specific disease states in the SABiosciences PCR array list (QIAGEN; www.sabiosciences.com) for Mus musculus. The numbers in the Y-axis represent number of genes in each category which are indicated on the X-axis.
